# GRB2 Nucleates T Cell Receptor-Mediated LAT Clusters That Control PLC-γ1 Activation and Cytokine Production

**DOI:** 10.3389/fimmu.2015.00141

**Published:** 2015-03-30

**Authors:** Mahmood Yousif Bilal, Jon C. D. Houtman

**Affiliations:** ^1^Interdisciplinary Graduate Program in Immunology, University of Iowa, Iowa City, IA, USA; ^2^Department of Microbiology, Carver College of Medicine, University of Iowa, Iowa City, IA, USA

**Keywords:** GRB2, TCR, cytokines, clustering, PLC-γ1

## Abstract

GRB2 is a ubiquitously expressed adaptor protein required for signaling downstream of multiple receptors. To address the role of GRB2 in receptor-mediated signaling, the expression of GRB2 was suppressed in human CD4+ T cells and its role downstream of the T cell receptor (TCR) was examined. Interestingly, GRB2 deficient T cells had enhanced signaling from complexes containing the TCR. However, GRB2 deficient T cells had substantially reduced production of IL-2 and IFN-γ. This defect was attributed to diminished formation of linker for activation of T cells (LAT) signaling clusters, which resulted in reduced MAP kinase activation, calcium flux, and PLC-γ1 recruitment to LAT signaling clusters. Add back of wild-type GRB2, but not a novel N-terminal SH3 domain mutant, rescued LAT microcluster formation, calcium mobilization, and cytokine release, providing the first direct evidence that GRB2, and its ability to bind to SH3 domain ligands, is required for establishing LAT microclusters. Our data demonstrate that the ability of GRB2 to facilitate protein clusters is equally important in regulating TCR-mediated functions as its capacity to recruit effector proteins. This highlights that GRB2 regulates signaling downstream of adaptors and receptors by both recruiting effector proteins and regulating the formation of signaling complexes.

## Introduction

The ubiquitously expressed adaptor protein GRB2 facilitates the assembly of multiprotein signaling complexes at activated receptors and signaling proteins. GRB2 and its homologs, GRB2 related adaptor protein (GRAP) and GRB2-related adaptor protein downstream of SHC (GADS), are composed of a central Src homology 2 (SH2) domain flanked by two Src homology 3 (SH3) domains (1–4). The SH2 domain binds specific phospho–tyrosyl residues present on signaling proteins such as growth factor receptors, insulin receptor substrate (IRS) proteins, and the hematopoietic specific adaptor protein linker for activation of T cells (LAT) (4–6). The SH3 domains of GRB2 bind proline-containing sequences on intracellular ligands that drive further downstream signaling. GRB2-mediated complexes regulate cell cycle progression, motility, angiogenesis, and differentiation of nearly all cell types (3, 7, 8). Due to its role in propagating signaling pathways downstream of numerous receptors, dysregulated GRB2-mediated signaling complexes has been linked to oncogenesis, autoimmunity, diabetes, and cardiovascular disease (3, 7, 8).

One physiological system dependent on GRB2 function is the activation of CD4+ T cells by the T cell receptor (TCR). Ligation of the TCR by peptide antigen bound major histocompatibility complexes (pMHC) on antigen presenting cells is the primary signal required for T cell activation, differentiation, and proliferation (9, 10). Within seconds after TCR ligation, multiple kinases are activated that facilitate the phosphorylation of the adaptor protein LAT (4, 11, 12). GRB2 and its homologs directly bind phosphorylated tyrosines at LAT, while simultaneously recruiting several proline rich ligands to this complex. The SH3 domain ligands for GRB2 include the E3 ubiquitin ligase CBL and the guanine nucleotide exchange factor SOS1, which modulates the activation of the MAP kinases ERK1 and ERK2 (12, 13). In addition, TCR-induced cytoskeletal arrangement and phosphatidylinositide 3-kinases (PI3K) activation is mediated through the localization of GADS and its SH3 domain ligand, SLP-76, to the LAT complex (9, 12, 14). Finally, LAT phosphorylation is critical for the recruitment and activation of phospholipase-γ1 (PLC-γ1), which directly modulates calcium influx and the activation of PKC isoforms (15).

In addition to its ability to recruit effector proteins, GRB2 may also regulate the formation of large, megadalton sized LAT clusters. All three GRB2 binding sites on LAT are required for the formation of LAT signaling clusters, and reduced dimerization of GRB2 via the overexpression of mutant SH3 domain ligands inhibits the formation of LAT clusters (4, 12, 15, 16). However, the LAT mutations and overexpression of SH3 domain ligands will also inhibit the function of GRAP and GADS. In fact, the dimerization of SLP-76 by its SH2 domain ligand ADAP has been suggested to facilitate the clustering of SLP-76 and LAT. Thus, whether GRB2 alone or the combined function of GRB2, GRAP, and/or GADS is critical for the formation of LAT clusters has not been directly demonstrated. In addition, it is unknown if LAT phosphorylation alone is sufficient for PLC-γ1 activation or if the formation of LAT microclusters is required for the recruitment of PLC-γ1 to the LAT complex. Thus, GRB2 may have both a signaling role, by recruiting ligands to the LAT complex, and a structural role in driving clustering of LAT, but the relative contribution of these events to function downstream of the TCR or other receptors is unknown.

Although GRB2 clearly regulates TCR-mediated signaling, its exact function downstream of this receptor is unknown. Recent studies utilizing GRB2 conditional knockout (KO) or haplo-deficiency in murine thymocytes demonstrated reduced proximal signaling and normal ERK1/ERK2 activation (17, 18). However, due to developmental issues, cells from these mice may have signaling defects arising from impaired development and not the role of these proteins in mature T cells. Also, human T cells with reduced GRB2 expression have normal TCR-induced ERK1/ERK2 activation but no other functions have been assessed in these cells (19). Thus, the full functions of GRB2 in mediating the different signaling pathways in mature human T cells are unclear.

In this study, we have developed effective recombinant microRNAs that suppress the expression of GRB2. We observed that GRB2 negatively controls early TCR signaling complexes. We also demonstrate for the first time that GRB2 alone regulates the formation of LAT microclusters, which drives PLC-γ1 recruitment to LAT, calcium influx, and cytokine release in mature human T cells. We have identified novel roles for GRB2 in mediating LAT signaling complexes and subsequent downstream signaling in mature human CD4+ T cells. Our studies also provide novel insight into how the ability of GRB2 to control effector protein recruitment and the formation of large signaling clusters is critical for the induction of PLC-γ1 and calcium signaling downstream of other receptors and adaptor proteins.

## Materials and Methods

### Isolation and growth of human CD4+ peripheral blood T cells

Peripheral blood mononuclear cells (PBMC) were first isolated from whole blood of healthy donors using leukocyte reduction system (LRS) cones as previously described (20). The blood donors consented for blood donation at the DeGowin Blood Center at the University of Iowa and to allow blood cells not used for transfusion to be used for research at the University of Iowa. The consent process and consent documents for these donors have been approved by the IRB for the University of Iowa. Because all cells used in these studies were obtained from normally discarded products, the donors approved for the use of their cells in research projects and the donors were completely de-identified; these studies were exempt from further IRB approval. Primary human CD4+ T cells were negatively selected using CD4+ T cell isolation kit II (Miltenyi Biotec), resulting in >95% purity. Isolated CD4+ T cells were then activated with anti-CD3 (OKT3, BioLegend) and anti-CD28 (CD28.2, BioLegend) crosslinked to magnetic Dyna beads (Invitrogen) in the presence of 100 U/mL IL-2. Cells were cultured for 3 days at 37°C and 5% CO_2_ in complete RPMI 1640 (RPMI media supplemented with 10% FBS, 50 U/mL penicillin, 50 μg/mL streptomycin, and 2 mM l-glutamine) (Gibco). Prior to human primary CD4+ T cell stimulation, cells were rested for 1 day in complete RPMI without magnetic beads or IL-2.

### Growth and stimulation of HuT78 T cell lines

HuT78 CD4+ T cell lines were cultured at 37°C and 5% CO_2_ in complete IMDM media (IMDM media supplemented with 20% FBS, 50 U/mL penicillin, 50 μg/mL streptomycin, and 2 mM l-glutamine) (Gibco). Cell lines transduced with retro or lentiviruses containing GRB2 or luciferase (LUC) micro/shRNAs and add-back GRB2 proteins were kept in selection with 2 μg/mL puromycin (CAS 58-58-2, Santa Cruz). Cell lines were grown to a concentration of 2–5 × 10^5^ cells/mL prior to stimulations, and then washed in un-supplemented RPMI 1640 (Gibco). They were then resuspended in un-supplemented RPMI 1640 at 5 × 10^7^ cells/mL and incubated for 10 min at 37°C. The cells were stimulated with soluble 2 μg/mL anti-CD3 (OKT3, BioLegend) for various time points and then lysed with the addition of fourfold excess of hot 2X lysis buffer (20 mM Tris pH 8.0, 2 mM EDTA, 2 mM Na_3_VO_4_, 20 mM DTT, 2% SDS, and 20% glycerol). Lysates were then heated to 95°C for 4 min and sonicated to reduce viscosity.

### Vectors and microRNA/shRNA design

#### Vectors

Murine stem cell virus (MSCV)-LTRmiR30-PIG (LMP) retroviral expression vector and VSV-G were a kind gift from Dr. Bruce Hostager. Lentiviral pLK4 vectors were donated by Dr. Stephen Bunnell. pLKO.1 lentiviruses were obtained from Addgene (plasmid 8453) (21). Packaging plasmids pCL-Eco and Pax2 were obtained from Drs. John Colgan and Dawn Quelle, respectively.

MicroRNA/shRNA targeting sequences against human GRB2 (NM_002086.4) were developed, utilizing a siRNA/shRNA based algorithm formulated by Dr. Sachidanandam and coworkers (http://katahdin.cshl.edu). The following sequences produced >90% suppression and was selected as the primary hairpin for targeting GRB2 in human T cells in this study (GRB2-7 sense: 5′ AGCAGAAGAAATGCTTAGCAAA 3′, anti-sense: 5′ TTTGCTAAGCATTTCTTCTGCC 3′). The following sequence produced >80% suppression and was selected as an alternative GRB2 microRNA (GRB2-10 sense: 5′ CCGGCTTCATTCCCAAGAACTA 3′, anti-sense: 5′ TAGTTCTTGGGAATGAAGCCGT′).

#### Hairpin delivery into HuT78 and primary human CD4+ T cells

HuT78 T cells were transduced with LMP retroviruses carrying microRNAs with the exception of knockdown/add-back experiments where lentiviruses carrying shRNAs were utilized. This is due to the inefficiency of lentiviral hU6 promotor in driving microRNA-induced suppression. Next, because retroviral LMP vectors carrying microRNAs are substantially less efficient at infecting human primary CD4+ T cells, YFP-positive pLK4 lentiviral vectors carrying shRNAs were used for their transduction. Sequences from pLKO.1 vectors containing the U6 promoter driving GRB2 or LUC shRNAs were amplified and cloned into pLK4 lentiviral vectors using the following primers (F hU6-*Spe*I: 5′ CAACCAACTAGTGAGGGCCTATTTCCCATGATTCCTTCATATTTGC 3′, RGRB2-*Pvu*I: 5′ TGGTTGCGATCGAAAAAAGGCAGAAGAAATGCTTAGCAAATACATCTG 3′, RLUC-*Pvu*I: 5′ TGGTTGCGATCGAAAAAATGGCTCCCGCTGAATTGGAATCTACATCT 3′).

#### shRNA-resistant GRB2 – wild-type and mutant add-back

To generate a wild-type GRB2 that is resistant to shRNA mediated knockdown, GRB2 cDNA was mutated using primers containing shRNA-sense bases to produce wild-type GRB2 with a different nucleotide sequence, but with an identical amino acid sequence. The following primers were used:

Wild-type GRB2 → Wild-type shRNA-Resistant GRB2 (F: 5′ GGCAAAATCCCCAGAGCCAAGGCGGAGGAGATGCTGAGTAAGCAGCGGCACGATGGGGCC 3′, R: 5′ GGCCCCATCGTGCCGCTGCTTACTCAGCATCTCCTCCGCCTTGGCTCTGGGGATTTTGCC 3′).

KVLN → AAAA mutation primer (F: 5′ TTCAAAAGGGGGGACATCCTCGCGGCTGCGGCCGAAGAATGTGATCAGAAC 3′, R: 5′ GTTCTGATCACATTCTTCGGCCGCAGCCGCGAGGATGTCCCCCCTTTTGAA 3′).

Wild-type or KVLN shRNA-resistant GRB2 constructs were then amplified and cloned into pLK4 vectors containing U6-GRB2 shRNA from above using the following primers (F-GRB2cDNA-*Age*I: 5′ CAGAATACCGGTCGCCACCATGGAAGCCATCGCCAAATA 3′, R-GRB2cDNA-*Not*I: 5′ CAGAATGCGGCCGCAAGGTATTAGACGTTCCGGTTCACG 3′).

### 293T calcium phosphate transfections and viral production

To produce pseudo-typed retro and lentiviral particles, the microRNA/shRNA containing vectors (LMP, pLK4) were transfected into 293T cells along with VSV-G envelope and packaging vectors pCL-Eco and Pax2 for LMP and pLK4, respectively. About 18–24 h prior to infection, 3 × 10^6^ 293T cells were seeded in 10 cm culture dishes at 37°C and 5% CO_2_ in complete DMEM media (DMEM media supplemented with 10% FBS, 50 U/mL penicillin, 50 μg/mL streptomycin, 1X MEM NEAA, and 2 mM l-glutamine) (Gibco). On the day of transfection, the media was replaced with 5 mL of fresh complete DMEM.

293T cells were transfected utilizing calcium phosphate method. Briefly, plasmids (LMP or pLK4 15 μg, pCL-Eco or Pax2 10 μg, VSV-G 7.5 μg) were mixed together in 0.5 mL of 1X HBS buffer (Hepes free acid 21 mM, NaCl 137 mM, D+ Glucose monohydrate 5 mM, KCl 50 mM, Na_2_HPO_4_ 0.35 mM, pH 7.5). Next, 30 μL of 2.5M CaCl_2_ was added drop-wise to the HBS-DNA solution, mixed immediately, and incubated for 30 min at room temperature. The HBS-DNA-CaCl_2_ mix was then added drop-wise around the plate of 293T cells and then incubated for 16–18 h. Transfection media was replaced with fresh media, and viral containing supernatant was subsequently harvested, and filtered through 0.45 μm Durapore Millex (Millipore) filters every 24 h for 2 days. Viral supernatants were divided into round-bottom polycarbonate high-speed tubes (Nalgene-Oak ridge). The tubes were centrifuged at 4°C for 1.5 h, 48,000 × g in Sorvall RC6-Plus centrifuge (SS-34 Rotor). Viral pellets were resuspended in complete RPMI media for subsequent transductions.

### Transduction of human T cell lines and primary CD4+ T cells

For HuT78 T cell transductions, 5 × 10^5^ cells were incubated in 1–1.5 mL of concentrated viral supernatant in 25 cm^2^ flasks in the presence of 8 μg/mL Hexadimethrine bromide (Sigma Aldrich) with periodical mixing. After 48 h, HuT78 T cells were resuspended in fresh complete IMDM with 0.5 μg/mL of puromycin and cells were allowed to expand. Puromycin was then increased gradually to 2 μg/mL after initial cell expansion.

For primary human CD4+ T cell transductions, purified cells were activated for 1 day as above, and then 10–20 × 10^6^ cells were incubated in 1–1.5 mL of concentrated viral supernatant in 25 cm^2^ flasks in the presence of 8 μg/mL Hexadimethrine bromide (Sigma Aldrich) with periodical mixing. IL-2 along with beads containing anti-CD3 and anti-CD28 were left during the transduction. About 1.5 mL of complete RPMI media, Hexadimethrine bromide, and IL-2 were added to the cells every 24 h. After 72–96 h, cells were resuspended in fresh complete RPMI without the beads or IL-2. Due to the short lifetime of primary CD4+, YFP+ cells were instead sorted using Becton Dickinson Aria II (Flow Cytometry Facility, University of Iowa) to obtain a population (>98%) of cells expressing GRB2 or LUC shRNA.

### Cytokine detection and RT-PCR

Cells were washed in complete RPMI 1640, and then resuspended at 2–5 × 10^5^ cells/mL. Cells were stimulated by adding 0.5 mL of cell suspension to 24 well plates coated with various concentrations of anti-CD3 for 24 h, or with 200 nM phorbol 12-myristate 13-acetate (PMA), and 1 μM ionomycin for 4.5 h. IL-2 and IFN-γ protein concentrations in the culture supernatants were measured using standard TMB ELISA utilizing a spectrophotometric plate reader with a reading absorbance at 450 nm. For RT-PCR, RNA was extracted from stimulated cells using Qiagen’s Rneasy RNA extraction kit per manufacturer’s protocol. The RT-PCR reaction was run using SuperScript III One-Step RT-PCR (Invitrogen). PCR primers were designed and obtained using IDT’s primer quest (IL-2 F: 5′ CAGTGCACCTACTTCAAGTTCTA 3′, IL-2 R: CCTCCAGAGGTTTGAGTTCTTC, IFN-γ F: GTGGAGACCATCAAGGAAGAC, IFN-γ R: CAGGCAGGACAACCATTACT). The reaction was run for 29 cycles and quantified using ImageStudio Lite (Licor).

### Immunoblotting

Protein containing samples (equivalent of 5 × 10^6^ cells) were loaded onto a 4–15% precast Criterion polyacrylamide gel (Biorad). The separated proteins were transferred onto PVDF membranes (Millipore), and then blocked for 1 h at room temperature in a 1:1 1XPBS:SEA Block buffer (Thermo Scientific). The PVDF membranes were then incubated with primary antibodies against GRB2 (clone 23, Santa Cruz Biotechnology), LAT pY226 (clone J96-1238.58.93, BD Pharmingen), LAT pY132 (Genetex), SLP-76 pY128 (clone J141-668.36.58, BD Pharmingen), ERK1/ERK2 pY187/pT185 (Invitrogen), p38 pT180/pY182 (Cell Signaling), JNK pT183/pY195 (Cell Signaling), Akt pS473 (clone-14-6, Invitrogen), Src pY416 (Cell Signaling), pY783 PLC-γ1 (Cell signaling), PLC-γ1 (Cell Signaling), lymphocyte-specific protein tyrosine kinase (LCK) (Cell Signaling), pY (4G10, Millipore), Gsk3αβ pS21/9 (Cell Signaling), actin (clone C4, Millipore), or GAPDH (Meridian Life Sciences). Secondary antibodies conjugated to IRDye 800CW or IRDye 680 were diluted in SEA Block and incubated with the PVDF membranes for 30 min at room temperature. The membranes were then visualized using the Licor Odyssey Infrared detector.

### Immunoprecipitations

10 × 10^6^ HuT78 T cells were washed in un-supplemented RPMI and stimulated as above. The cells were then lysed using 800 μL of immunoprecipitation buffer (25 mM Tris pH 8.0, 150 mM NaCl, 1% Brij-97, 0.5% *n*-Octyl-β-d-glucopyranoside, 5 mM EDTA, 1 mM Na_3_VO_4_, complete protease inhibitor tablets). The cellular lysates were incubated on ice for 30 min and then centrifuged (13000 rpm, 10 min). The supernatants were pre-cleared by rotating for 30 min with protein A/G plus agarose (Santa Cruz Biotech), centrifuged, and the pre-cleared supernatants were incubated with protein A/G plus agarose with or without immunoprecipitation antibody overnight at 4°C. The beads were washed three times by centrifugation (5000 rpm, 2 min) in immunoprecipitation washing buffer (25 mM Tris pH 8.0, 150 mM NaCl, 5 mM EDTA, complete protease inhibitor tablets). The immunoprecipitated proteins were eluted by incubating with 2X lysis buffer at 95°C for 4 min.

### Immunoblot analysis

Densitometric analysis of protein bands was determined using Odyssey’s v3.0 software. Normalization of phospho specific proteins to pan forms may give misleading results due to the inability of pan antibodies to detect phosphorylated forms and steric hindrance that can occur when two antibodies bind to the same small protein. Thus, phosphorylated or total forms of proteins were normalized to actin as previously described (20, 22). The following formula was utilized for normalization and quantification of the immunoblots, cytokine release, and RT-PCR:

1. Normalized data point to actin intensities (NP) = raw band intensity ÷ raw actin intensity.
2. Percent of maximum of LUC stimulation (as indicated) = (NP time point-NP 0 time point of control LUC) ÷ (NP max time point of control LUC-NP 0 time point of control LUC).

### Flow cytometry

1 × 10^6^ cells were washed in FACS buffer (PBS, 10% FBS, and 0.05% sodium azide), and then resuspended in FACS buffer to a concentration of 1 × 10^6^ cells/mL. The mean fluorescence intensity (MFI) of each sample was obtained using Accuri C6 flow cytometer.

### Calcium influx

1 × 10^6^ HuT78 T cells were washed and resuspended in 1 mL RPMI 1640 without phenol-red (Gibco). About 5 μL of 1 mM Fluo-4 AM (Life Technologies), and 10 μL of 250 mM probenecid (Life Technologies) were added to the samples, and then incubated at 37°C and 5% CO_2_ for 45 min. Cells were washed twice in RPMI without phenol-red and then resuspended at 1 × 10^6^ cells/mL in RPMI-probenecid, and left on ice for 15 min. Cells were incubated at 37°C 5 min prior to stimulations. Cells were run un-stimulated for 35 s, and then stimulated with 5 μg/mL anti-CD3 for 6–7 min. The MFI of each sample was obtained using Accuri C6 flow cytometer. Statistical analysis per stimulation was calculated based on 10 live cell events per tenth of a second (i.e., 100 cells/s). Groups were graphed based on un-stimulated fold increase.

For assays requiring calcium free media, cells were prepared as above. After incubation in RPMI on ice for 15 min, cells were washed in warm HBSS without phenol-red (Gibco), with or without calcium. Cells were then stimulated with anti-CD3 for 6 min.

### Total internal reflection fluorescence microscope

Cell membrane imaging was performed using Leica AM TIRF MC imaging system as previously reported (20, 23). Briefly, 2.5 × 10^5^ HuT78 or primary CD4+ T cells were seeded onto glass chamber slides (LabTek II, Nalgene Nunc International) that were coated with 10 μg/mL anti-CD3. Cells were activated for the indicated times and then fixed with 3% paraformaldehyde-PBS solution for 30 min. The cells were then permeabilized with 0.25% TritonX-100 in PBS for 5 min and washed in PBS, blocked with SEA block, and stained overnight with the indicated antibodies. Cells were then incubated with secondary antibodies (Alexa 488 goat anti-mouse IgG1-BioLegend, Alexa 568 goat anti-mouse IgG1-Invitrogen) for 2 h. Images were acquired using Leica AF software and processed using Adobe Photoshop and ImageJ software. All images were captured using 100X oil objective.

Analysis of LAT cluster intensities was performed by drawing one line per cell through original images and performing “Plot Profile” analysis on ImageJ. At least 20 cells from three independent experiments (greater than 60 cells/group) were analyzed, compiled, and graphed as pixel-intensity plots. For 3D cluster analysis, ImageJ’s “Maximum Entropy Threshold” segmentation analysis was performed on 8-bit grayscale images, and graphed utilizing ImageJ’s “Interactive 3D Surface Plot”.

### Statistical analysis

Analysis of immunoblots, RT-PCR, and cytokine assays were performed in Graphpad prism software using two-tailed *t*-test assuming equal variance. Analysis of calcium influx data was performed using Microsoft Excel using a two-tailed *t*-test. Levels of significance *p* < 0.05 and *p* < 0.005 are presented as ∗ and ∗∗, respectively.

## Results

### Reduced expression of GRB2 through microRNA and shRNA-mediated suppression

**Figure 1:**
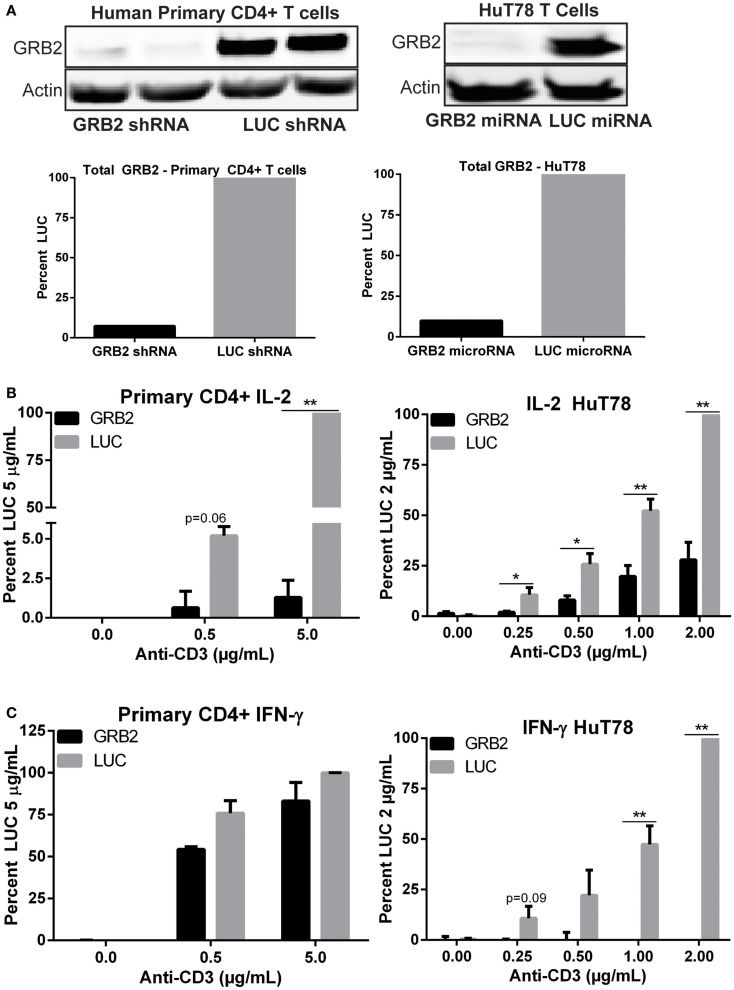
**GRB2 deficient human CD4+ T cells exhibit substantial reduction in TCR-induced cytokines release**. **(A)** Top: expression of GRB2 and actin in HuT78 and human primary CD4+ YFP+ T cells carrying GRB2 or luciferase (LUC) microRNAs and shRNAs, respectively. Bottom: Quantification of GRB2 suppression in primary human CD4+ and HuT78 T cells. **(B,C)** IL-2 and IFN-γ production in GRB2 deficient and LUC HuT78 T cells or YFP+ primary human CD4+ T cells stimulated with plate bound anti-CD3 for 24 h. The mean normalized value ± SEM of seven independent experiments (HuT78 T cells) or three different donors (primary CD4+ T cells) is shown.

We cloned potential microRNA targeting sequences for GRB2 into a modified form of the human miR30 microRNA in viral packaging vectors that have a gene for YFP to identify transduced cells. The most efficacious sequence produced >90% suppression of GRB2 expression in both CD4+ HuT78 T cells and YFP+ primary human CD4+ T cells from normal donors (Figure 1A and Figure S1A in Supplementary Material). Interestingly, the hU6 promoter in the lentiviral constructs was only efficient if the targeting sequence was inserted as a shRNA instead of microRNA (data not shown). The structural difference between the microRNA and the shRNA is that the microRNA contains flanking miR30 sequences while in the shRNA these sequences were removed, but the GRB2 targeting sequence is identical in both these suppressive RNAs. However, there was no large difference in the effectiveness of each type of targeting sequence, since our studies in HuT78 T cell line performed utilizing both miR30 and shRNA constructs had identical results (data not shown). To confirm that there were no off target effects in signaling pathways downstream of the TCR, the expression of various signaling proteins was examined. Surface TCR levels between GRB2 and control cells transduced with a microRNA targeting firefly LUC displayed similar expression (Figure S1B in Supplementary Material). In addition, there was no difference in total protein expression for LAT, PLC-γ1, SOS1, LCK, GADS, AKT, SLP-76, and PI3K between GRB2 deficient and LUC cells (Figures S1C–E in Supplementary Material).

### GRB2 is required for TCR-induced cytokine production in human CD4+ T cells

**Figure 2:**
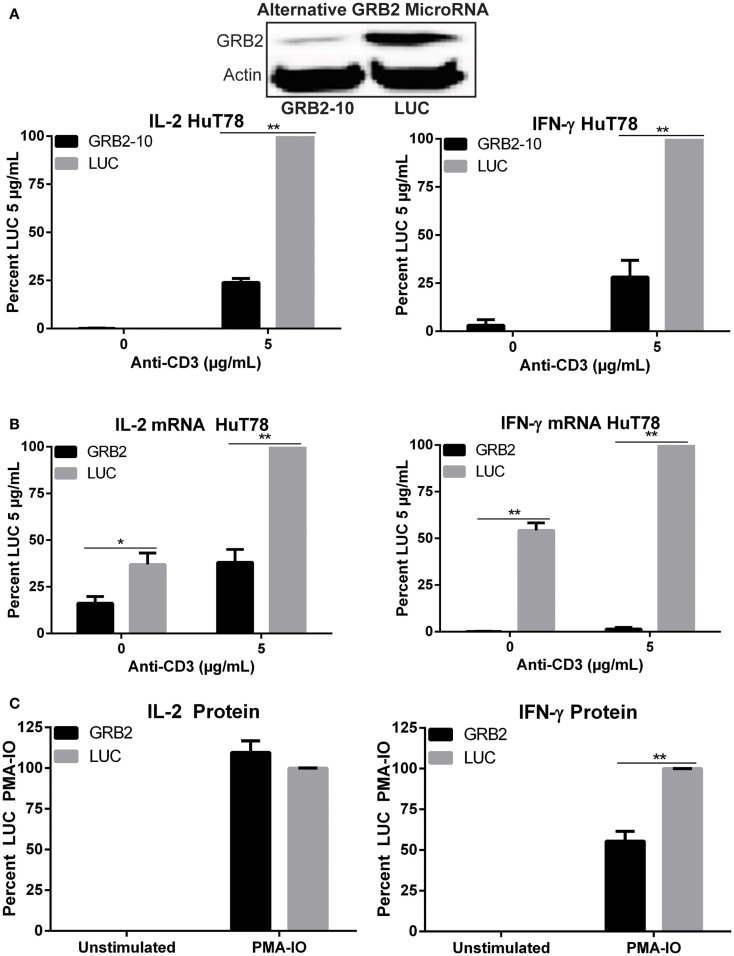
**Impaired TCR-induced transcriptional activity and PMA-IO mediated cytokine release in GRB2 deficient HuT78 T cells**. **(A)** HuT78 T cells carrying an alternative GRB2 (GRB2-10) or LUC microRNA were stimulated with 5 μg/mL plate-bound anti-CD3 for 24 h and the supernatants were probed for IL-2 and IFN-γ via ELISA. The mean normalized value ± SEM from three independent experiments is shown. **(B)** IL-2 and IFN-γ mRNA expression in HuT78 T cells carrying primary GRB2 or LUC microRNAs stimulated with 5 μg/mL anti-CD3 for 24 h. The mean normalized value ± SEM from three independent experiments is shown. **(C)** IL-2 and IFN-γ protein release in HuT78 T cells stimulated with PMA-IO for 4.5 h. The mean normalized value ± SEM from three independent experiments is shown.

The role of GRB2 in controlling downstream T cell functions has not been tested extensively. To address this issue, we examined the ability of GRB2 deficient T cells to secrete the crucial immunomodulatory cytokines, IL-2, and IFN-γ. Both IL-2 and IFN-γ production were significantly reduced in GRB2 deficient HuT78 T cells compared to control cells (Figures 1B,C). Similarly, GRB2 deficient primary human CD4+ T cells exhibited a significant reduction of IL-2 release, but had comparable IFN-γ secretion (Figures 1B,C). The contrasting effects on IFN-γ release may be due to the strong priming received via CD3 and CD28 prior to GRB2 knockdown. HuT78 T cells carrying an alternative GRB2 microRNA also displayed impaired IL-2 and IFN-γ production (Figure 2A), showing that the suppression of GRB2 expression, and not specific off-target effects, are driving reduced cytokine release. These results were confirmed at the mRNA levels as transcripts of IL-2 and IFN-γ were diminished in GRB2 deficient cells relative to control HuT78 T cells (Figure 2B). Interestingly, basal levels of IFN-γ mRNA were markedly reduced in un-activated GRB2 deficient cells compared to control (Figure 2B).

To assess if the impairment in cytokine production occurred due to defects at the TCR or LAT complexes, GRB2 deficient cells were treated with PMA and ionomycin (IO), a strong T cell activator that bypasses the TCR and LAT complexes. Interestingly, upon PMA-IO stimulation, GRB2 deficient cells were able to secrete similar levels of IL-2 and a modest amount of IFN-γ compared to control LUC HuT78 T cells (Figure 2C). Overall, these results indicate a requirement for GRB2 in driving cytokine production in human CD4+ T cells, and that the defects in TCR-induced cytokine production in GRB2 deficient cells are due to alterations in proximal signaling at the TCR and LAT complexes.

### GRB2 deficient cells display enhanced proximal TCR signaling

**Figure 3:**
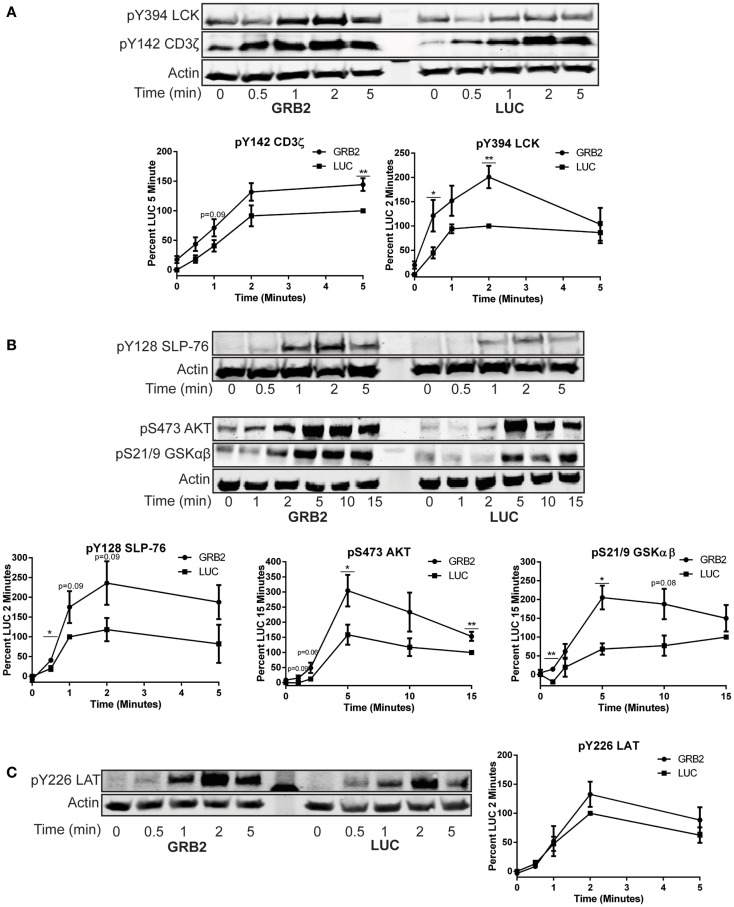
**GRB2 deficient cells display enhanced proximal TCR signaling**. The phosphorylation of proteins in GRB2 deficient or control HuT78 T cells stimulated with 2 μg/mL soluble anti-CD3 was detected by immunoblotting using antibodies against **(A)** pY416 Src (LCK) *n* = 6, pY142 CD3ζ chain *n* = 5. **(B)** pY128 SLP-76 *n* = 6, pSer473 AKT *n* = 7, pSer21/9 GSKαβ *n* = 3. **(C)** pY226 LAT *n* = 5. The levels of phosphorylation were normalized to actin expression and graphed as mean percentage phosphorylation of LUC ± SEM for each time point.

The previous data shows that GRB2 is required for optimal cytokine production in human CD4+ T cells. To determine how GRB2 controls signaling pathways downstream of the TCR, we examined the effects of GRB2 deficiency on TCR-mediated signaling. TCR ligation induces the activation and/or recruitment of the Src family kinase LCK, which then phosphorylates proteins at the TCR complex including ZAP-70 and the TCR ζ chain (10). Enzymatically active ZAP-70 phosphorylates and activates essential signaling proteins at the TCR/LAT complexes including LAT and SLP-76 (10, 24). In contrast to our expectations based on the cytokine release data, GRB2 deficient HuT78 T cells exhibited augmented tyrosine phosphorylation in the activation site of LCK (Figure 3A). As expected by the enhanced LCK activation, phosphorylation levels of ZAP-70 and TCR ζ chain were also increased in the absence of GRB2 (Figure 3A and Figure S2A in Supplementary Material). Additionally, SLP-76 phosphorylation at Y128 was enhanced in GRB2 deficient cells (Figure 3B). We also observed increased phosphorylation of AKT and GSKαβ, both of which are dependent on the recruitment of PI3K by SLP-76 to the LAT signaling complex (Figure 3B) (14). These findings are supported by the observation that GRB2 deficient cells, HuT78 T cells, have enhanced total TCR-induced tyrosine phosphorylation of the above mentioned proximal proteins (Figure S2A in Supplementary Material). These results indicate that GRB2 functions as a negative regulator at the TCR complex.

### LAT phosphorylation on the GRB2 binding site (Y226) is not impaired in the absence of GRB2

Previous studies have demonstrated that GRB2 family of adaptors bind phosphorylated LAT Y171, Y191, and Y226, thereby driving essential TCR-mediated signaling (12, 15, 25). It is possible that GRB2 binding protects these sites on LAT from being dephosphorylated by specific phosphatases. However, the increased function of ZAP-70, the kinase for LAT, could result in enhanced LAT phosphorylation. To dissect whether GRB2 deficiency alters LAT phosphorylation on GRB2 binding site after TCR stimulation, GRB2 and LUC HuT78 T cells were stimulated and probed for total phosphorylated LAT and Y226. Compared to the GRB2 homolog, GADS, pY226 on LAT has substantially more affinity for GRB2 binding *in vivo* (25). Additionally, monoclonal antibodies against pY226 are more specific and have no variation between batches compared to polyclonal pY191 antibodies (data not shown). Interestingly, we found that both total and LAT Y226 phosphorylation are not affected by the absence of GRB2 (Figure 3C and Figure S2A in Supplementary Material). These data suggest that GRB2 is not required for phosphorylation of LAT at Y226.

### GRB2 is required for optimal TCR-induced MAP kinase activation

**Figure 4:**
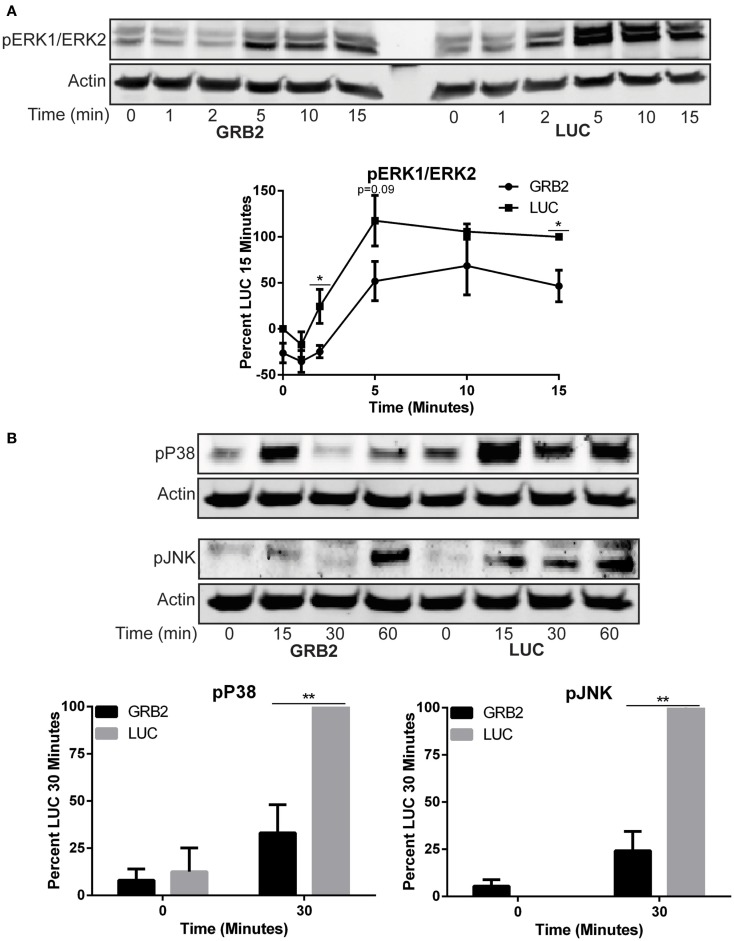
**Activity of MAP kinases, ERK1/ERK2, p38, and JNK is reduced in the absence of GRB2**. The phosphorylation of proteins in GRB2 deficient or control HuT78 T cells stimulated with 2 μg/mL soluble anti-CD3 was detected by immunoblotting using antibodies against **(A)** pY187/pT185 ERK1/ERK2 *n* = 5, pT180/pY182 p38 *n* = 5, **(B)** pT183/pY195 JNK *n* = 5. The levels of phosphorylation were normalized to actin expression and graphed as mean percentage phosphorylation of LUC ± SEM for each time point.

GRB2 is thought to facilitate the activation of ERK1/ERK2 in T cells by linking SOS1 to Ras at the cellular membrane (26). However, recent studies have challenged the requirement of the GRB2-SOS1 complex in driving full activation of TCR-induced ERK1/ERK2 (18, 19). The activation of p38 and JNK is mediated through small GTP binding proteins RAC-1, CDC42, and RHO, but the mechanisms for the activation of p38 and JNK upon TCR stimulation are not well characterized (27–29). Similar to previous studies, we observed that phosphorylation of ERK1/ERK2 was moderately reduced, but not completely suppressed, 10–15 min after activation when GRB2 expression is suppressed in HuT78 T cells (Figure 4A). The activation of p38 and JNK were substantially, but not completely, reduced in the absence of GRB2 (Figure 4B). Our results corroborate earlier findings indicating that GRB2 is needed for optimal activation of ERK1/ERK2 (17–19), and demonstrate that GRB2 is also essential for optimal TCR-induced p38 and JNK activation.

### GRB2 is essential for the activation and recruitment of PLC-γ1 to the LAT signalosome

**Figure 5:**
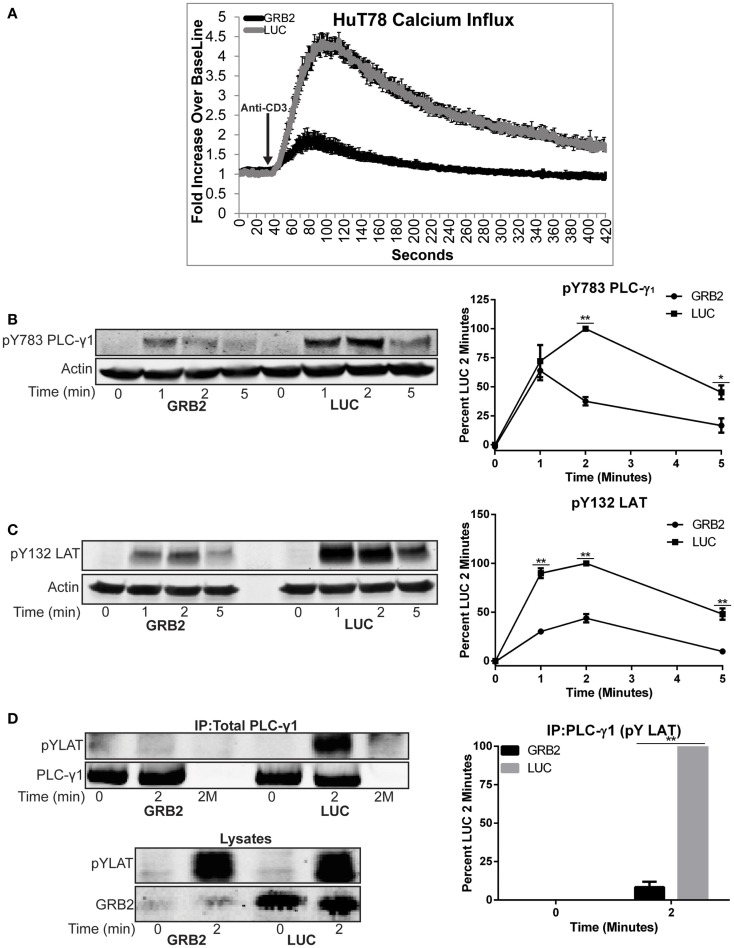
**GRB2 deficient cells have impaired TCR-induced calcium influx and recruitment of PLC-γ1 to the LAT complex**. **(A)** Calcium influx in GRB2 deficient or control HuT78 T cells stimulated with 5 μg/mL soluble anti-CD3. The data is shown as fold increase of average cellular fluorescent intensity over baseline average cellular fluorescent intensity ± SEM of four independent experiments. **(B)** The phosphorylation of PLC-γ1 in GRB2 deficient or control HuT78 T cells stimulated with 2 μg/mL soluble anti-CD3 was detected by immunoblotting using antibodies against pY783. The levels of phosphorylation of PLC-γ1 was normalized to actin expression and graphed as mean percentage phosphorylation of LUC ± SEM for each time point of four independent experiments. **(C)** GRB2 deficient or control HuT78 T cells were stimulated as in “**(B)**” and then the protein levels were detected using antibodies against pY132 LAT and actin. The levels of phosphorylation of Y132 was normalized to actin expression and graphed as mean percentage phosphorylation of LUC ± SEM of four replicates. **(D)** PLC-γ1 was immunoprecipitated from HuT78 T cells stimulated with 2 μg/mL soluble anti-CD3 for 2 min with anti-PLC-γ1 or no antibody control (2M) and the phosphorylation or binding of specific proteins was assessed by immunoblotting. Data shown is representative of four independent experiments.

After TCR ligation, LAT is rapidly phosphorylated, thereby allowing the recruitment of PLC-γ1 to the cellular membrane (12, 13, 23). PLC-γ1 is then activated through phosphorylation on Y783, resulting in enhanced calcium influx needed for cytokine production (10, 15). Because PLC-γ1 is recruited to LAT and the role it plays in cytokine production, we assessed if GRB2 deficient cells had impaired calcium influx. Interestingly, HuT78 T cells with reduced GRB2 expression had marked reduction in the peak levels of calcium influx and time to return to baseline calcium levels (Figure 5A). Stimulating GRB2 deficient cells in calcium-free media resulted in reduced release of internal calcium stores relative to control cells (Figure S2B in Supplementary Material), suggesting a defect in PLC-γ1 function.

We next examined if the phosphorylation and recruitment of PLC-γ1 to LAT was diminished in the absence of GRB2. GRB2 deficient cells had reduced phosphorylation of Y783 on PLC-γ1 compared to control HuT78 T cells (Figure 5B). We next hypothesized if the reduction of PLC-γ1 phosphorylation was due to impaired phosphorylation of LAT Y132, the PLC-γ1 binding site on LAT. In contrast to LAT Y226, but similar to Y783 on PLC-γ1, phosphorylation of LAT Y132 was impaired in the absence of GRB2 (Figures 3C and 5B,C). Next, because the phosphorylation levels of Y783 and Y132 on PLC-γ1 and LAT, respectively, did not correlate with the marked reduction of calcium mobilization, we asked if recruitment of PLC-γ1 to LAT was impaired in the absence of GRB2. Corresponding with the attenuated calcium flux, the interaction between PLC-γ1 and LAT was undetectable in GRB2 deficient T cells after TCR stimulation (Figure 5D). Interestingly, the latter occurred even as the total or Y226 LAT phosphorylation was similar or slightly increased in GRB2 deficient cells (Figures 3C and 5D). These data indicate that phosphorylation of LAT Y132 regulates the activation but not the stability of PLC-γ1 binding to the LAT complex. Overall, our results demonstrate that GRB2 is required for the optimal activation and recruitment of PLC-γ1 to the LAT complex and subsequent cytoplasmic calcium mobilization.

### GRB2 facilitates the nucleation of LAT microclusters

Upon TCR activation, multiprotein complexes at LAT are concentrated into signaling microclusters. Disruption of LAT signaling clusters through the mutation of LAT-oligomerizing ligand SOS1 attenuates TCR-induced calcium signaling (4, 16). Similarly, mutation of LAT tyrosines responsible for GRB2, GRAP, and GADS binding impairs LAT-induced signaling, including PLC-γ1 phosphorylation and calcium influx (4, 15). Because of these data, it was hypothesized that GRB2 is required for LAT clustering by recruiting SOS1 to LAT (4, 16). However, it is unclear if GRB2 alone is required for the formation of LAT clusters or if other homologs, such as GRAP, that can bind SOS1, may also have a role in LAT oligomerization (30). Additionally, it is unclear what large scale clustering of LAT does to facilitate downstream signaling from the LAT complex.

**Figure 6:**
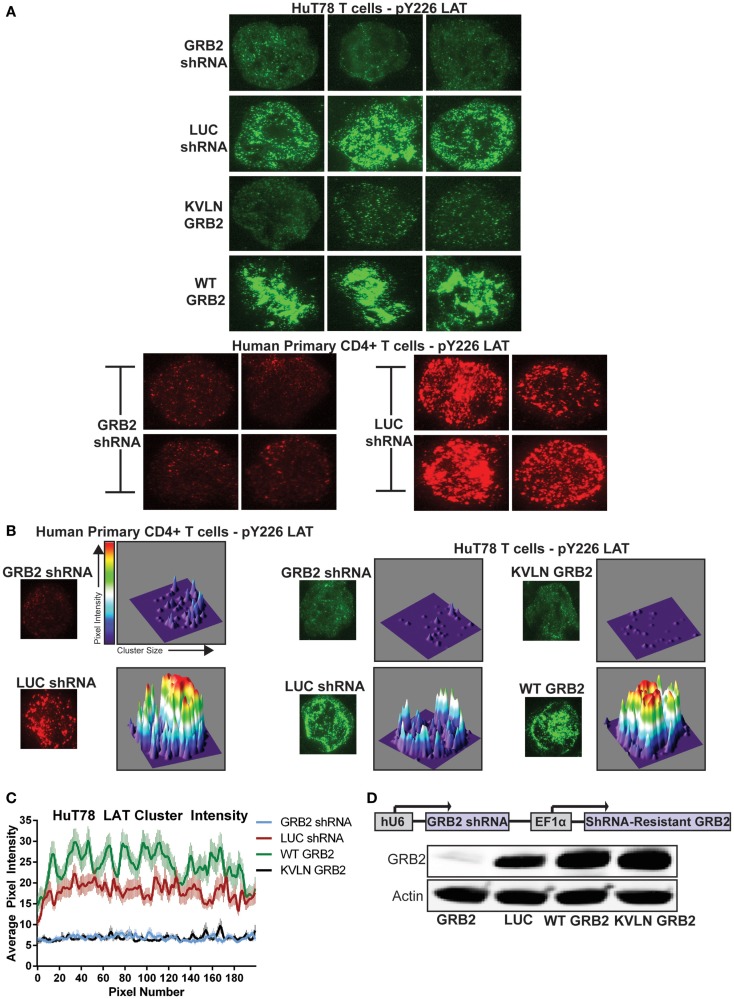
**GRB2 deficiency disrupts LAT microclusters**. **(A)** HuT78 or primary human CD4+ YFP+ T cells were stimulated for 5 min on anti-CD3 coated glass coverslips. LAT microclusters were visualized by immunofluorescent TIRF microscopy utilizing pY226 LAT antibody. **(B)** HuT78 or primary human CD4+ YFP+ T cells were stimulated as in **(A)**. For 3D visualization of microclusters, single cells were analyzed and graphed using ImageJ’s segmentation analysis and 3D interactive plot. **(C)** The average intensities of clusters across the axis of T cells containing GRB2 shRNA (*n* = 65), LUC shRNA (*n* = 60), wild-type GRB2 (*n* = 60), KVLN (*n* = 60) were quantified using ImageJ’s “Plot Profile” function. **(D)** Top: schematic diagram of GRB2 knockdown and shRNA-resistant protein reconstitution. Bottom: The expression of GRB2 in HuT78 T cells transduced with lentiviral constructs carrying LUC and GRB2 shRNA with or without re-expression of shRNA-resistant GRB2 proteins.

To test if GRB2 is required for the formation of LAT microclusters, we utilized high resolution total internal reflection fluorescence microscopy (TIRF). This method allows the visualization of T cell plasma membrane with a depth of up to 150 nm, which allows the imaging of LAT microclusters without background noise from the cell body. To this end, HuT78 T cells expressing GRB2 or control LUC shRNA were stimulated with anti-CD3 bound glass chambers and then probed with antibodies against phosphorylated LAT Y226, which we previously found to be phosphorylated equally in GRB2 sufficient and deficient HuT78 T cells (Figure 3C). A marked disruption of LAT oligomerization was observed in GRB2 deficient HuT78 and primary human CD4+ T cells compared to control cells (Figure 6A). Clustering algorithms with 3D graphical analysis demonstrate dense LAT clusters in control cells when compared to GRB2 deficient primary CD4+ and HuT78 T cells (Figure 6B). Additional quantification of LAT cluster signal intensity in multiple cells revealed significant reduction of the intensity of LAT clusters along the cell axis in GRB2 deficient HuT78 T cells when compared to control cells (Figure 6C and Figure S2C in Supplementary Material).

**Figure 7:**
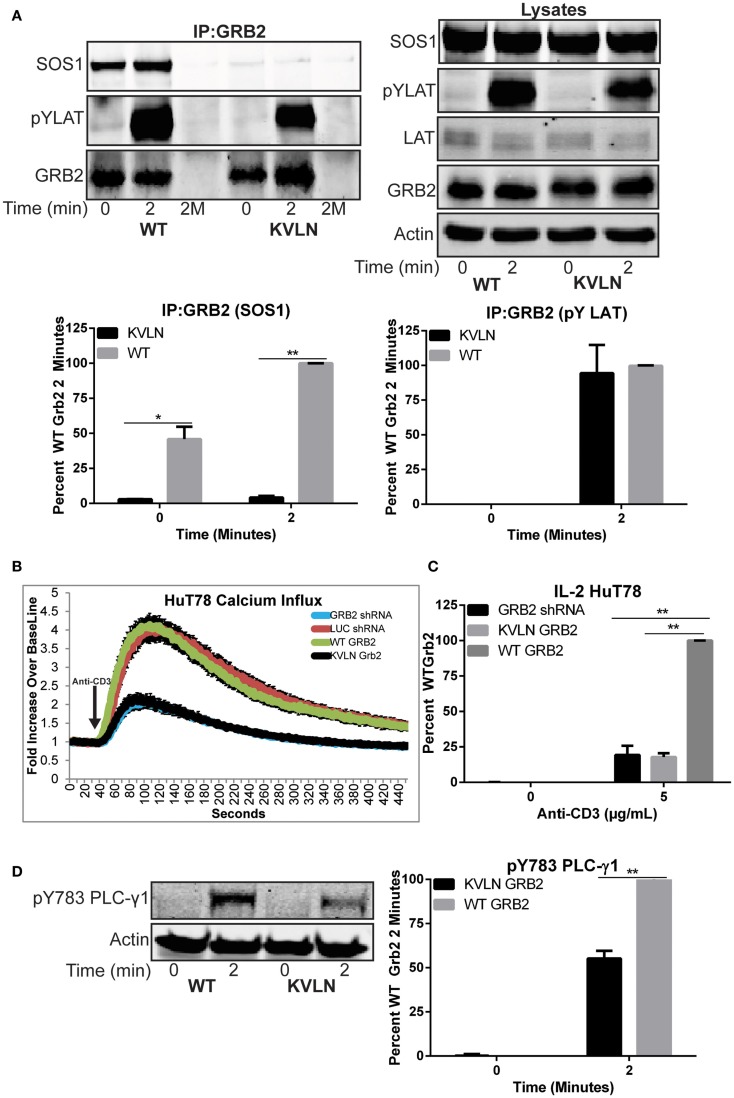
**Rescue of T cell function after reconstitution of wild-type but not KVLN GRB2**. **(A)** Top: GRB2 was immunoprecipitated from HuT78 T cells expressing wild-type or KVLN GRB2 stimulated for 2 min with 2 μg/mL soluble anti-CD3. The expression or phosphorylation of various proteins in the immunoprecipitates and lysates were assessed by immunoblotting. The data shown is a representative of three experiments. Bottom: quantification of GRB2:SOS1 and GRB2:pYLAT binding from GRB2 immunoprecipitation. **(B)** Calcium influx in HuT78 T cells transduced with lentiviral constructs carrying LUC and GRB2 shRNA with or without re-expression of shRNA-resistant GRB2 proteins stimulated with 5 μg/mL soluble anti-CD3. The data is shown as fold increase of average cellular fluorescent intensity over baseline average cellular fluorescent intensity ± SEM of four independent experiments. **(C)** IL-2 production in HuT78 T cells transduced with GRB2 shRNA with or without re-expression of shRNA-resistant GRB2 proteins stimulated with 5 μg/mL plate-bound anti-CD3 for 24 h. The mean normalized value ± SEM from three independent experiments is shown. **(D)** The phosphorylation of PLC-γ1 in HuT78 T cell lines expressing wild-type or KVLN GRB2 stimulated for 2 min soluble anti-CD3 was detected by immunoblotting using antibodies against PLC-γ1 pY783. The levels of phosphorylation of PLC-γ1 was normalized to actin expression and graphed as mean percentage phosphorylation of LUC ± SEM of three replicates.

We next examined if the ability of GRB2 to bind SH3 domain ligands is needed for LAT clustering. To accomplish this, we suppressed endogenous GRB2 and then re-expressed a shRNA-resistant wild-type GRB2 or an N-terminal SH3 domain (KVLN → AAAA) mutant of GRB2. The KVLN motif was identified due to its location around residues missing from the GRB2 crystal structure (residues 28–33) of the N-terminal SH3 domain of GRB2 (31). This suggested that a KVLN → AAAA mutation would inhibit the GRB2-SOS1 interaction. The shRNA-resistant wild-type and KVLN mutant could be expressed in HuT78 T cells where intrinsic GRB2 was substantially reduced (Figure 6D). As assessed by GRB2 immunoprecipitations, both wild-type and KVLN GRB2 interacted with phosphorylated LAT, but only wild-type GRB2 was able to bind to SOS1 (Figure 7A). This demonstrates that the KVLN mutant of GRB2 disrupts the interaction with SH3, but not SH2 domain ligands. Interestingly, LAT microclusters found in cells reconstituted with wild-type GRB2 were consistently denser and more intense when compared to control HuT78 T cells (Figures 6A–C). The latter is likely due to relative overexpression of GRB2 in reconstituted cells that leads to enhanced LAT aggregation compared to LUC control cells. Reconstitution of GRB2 N-terminal KVLN mutant in GRB2 deficient cells was unable to rescue the formation of LAT microclusters (Figures 6A–C). These data demonstrate the importance of the N-terminal SH3 domain of GRB2 in mediating LAT oligomerization. We then examined if reconstitution of clustering by GRB2 variants rescued T cell functions suppressed in GRB2 deficient T cells. GRB2 deficient cells reconstituted with wild-type GRB2 but not the KVLN mutant were able to recover calcium mobilization and cytokine production (Figures 7B,C) absent in GRB2 deficient T cells. HuT78 T cells reconstituted with KVLN mutant GRB2 had reduced PLC-γ1 phosphorylation when compared to cells containing wild-type GRB2 (Figure 7D). Overall, our results demonstrate that the binding of GRB2 to SH3 domain ligands is critical for the TCR-induced nucleation of large scale LAT microclusters, which ultimately drive subsequent PLC-γ1 phosphorylation, calcium influx, and cytokine production.

## Discussion

In the present study, we have potently silenced the expression of GRB2 in mature primary human CD4+ T cells and the HuT78 CD4+ T cell line. Surprisingly, our data demonstrates that GRB2 deficiency substantially enhances LCK, CD3ζ chain, ZAP-70, and SLP-76 phosphorylation (Figure 3 and Figure S2A in Supplementary Material), suggesting GRB2 negatively regulates the first signaling events at the TCR complex. Elucidating how GRB2 negatively controls proximal TCR signals is a massive effort beyond the scope of the present study. However, we hypothesize that GRB2 negatively controls TCR activation in two different manners. First, dimeric GRB2 inhibits the activation of the FGF receptor (FGFR), but upon tyrosine phosphorylation, dimerization is disrupted and FGFR inhibition is released (6). GRB2 associates with CD3ζ chain and ZAP-70 and has been localized by microscopy to the TCR complex (32–34). We propose that GRB2 could dimerize and interact with these molecules, thereby increasing the threshold of TCR activation. Second, GRB2 may recruit proline rich negative regulators to the TCR complex. This process may occur independently or through other mediators, such as the GRB2 binding protein SHC. In fact, SHC has been found to be associated with GRB2, the CD3ζ chain of the TCR, LCK, and ZAP-70 (32, 33, 35, 36). Thus, GRB2, alone or via SHC, may recruit negative regulators such as CBL, LYP, and FAK to interact with the TCR complex, thereby reducing CD3ζ, ZAP-70, or LCK activation (20, 37, 38). Collectively, these results demonstrate that GRB2 plays an important role in controlling the magnitude of early TCR signals.

In contrast to our studies, GRB2 deficient murine thymocytes had reduced TCR-induced phosphorylation of LCK and ZAP-70 (18). The discrepancy may originate from issues with T cell development or differences in signaling between murine thymocytes and human mature T cells (39). Indeed, GRB2 KO CD4+ T cells failed to up-regulate CD3 and down-modulate heat-stable antigen (HSA) (18). Thus, a failure to arrange critical TCR molecules during development likely leads to the diminished signaling deficiency observed in GRB2 KO thymocytes. Interestingly, GRB2 KO B cells display enhanced proximal signaling and calcium mobilization, but these cells do not form germinal centers (40, 41). Overall, these data suggest that GRB2 differentially controls signaling complexes in diverse cell types depending on available ligands.

We also observed that GRB2 is not absolutely required for the activation of ERK1/ERK2. This outcome was not surprising, as recent work demonstrated that ERK1/ERK2 activation was similar or even enhanced depending on the stimuli when both GRB2 and SOS1 expression were moderately suppressed in primary human T cells with synthetic siRNA (19). Likewise, GRB2 KO thymocytes had similar activation of ERK1/ERK2 compared to wild-type (18). In contrast and similar to murine thymocytes, p38 and JNK were markedly reduced in GRB2 deficient HuT78 T cells. The inability of GRB2 deficiency to inhibit ERK1/ERK2 activation can be linked to multiple factors. First, RasGRP rather than SOS1 may drive TCR-induced ERK1/ERK2 activation (19, 42, 43). Knockdown of RasGRP but not SOS1/2 altered ERK1/ERK2 activation (19), but both RasGRP and SOS1 are required for sustained ERK1/ERK2 activation in murine thymocytes and primary human CD4+ T cells (16, 43). Activation of Ras by RasGRP through the PLC-γ1/DAG pathways is possible in T cells (26). However, our data suggest that this pathway is defective due to reduced PLC-γ1 signaling in the absence of GRB2. This suggests a second mechanism where a compensatory adaptor protein such as the GRB2 homolog GRAP may produce redundant TCR-induced signaling, since GRAP can also bind to LAT and SOS1 (30).

**Figure 8:**
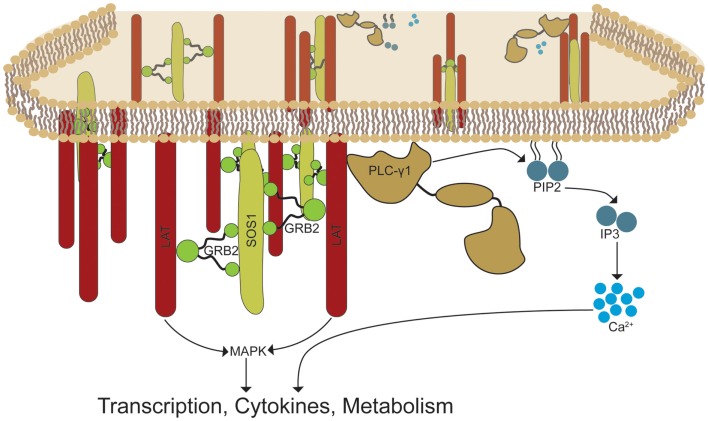
**GRB2-induced LAT oligomerization control the recruitment and activation of effector signaling complexes**. TCR activation induces the phosphorylation of LAT molecules by TCR-associated proximal kinases. GRB2 recruits SOS1 and other proline rich ligands to the LAT complex by linking its SH2 domain to the phospho-tyrosines at LAT. GRB2-SOS1 complexes create a signaling framework by linking multiple LAT proteins, thereby forming LAT signaling microclusters. The assembly of LAT clusters is critical for the recruitment and stability of signaling molecules such as PLC-γ1 and molecules required for MAP kinase activation. These events lead to essential signal transduction required for T cell effector function.

Our data demonstrate that GRB2 facilitates the nucleation of LAT microclusters in human T cells (Figure 8). Although it was previously hypothesized that GRB2 is essential for inducing LAT signaling clusters through the recruitment of SH3 domain ligands such as SOS1, SOS2, and CBL, this concept has not been directly demonstrated (4, 16). Since other SH3 domain containing proteins such as GADS and GRAP can be dimerized by SH3 domain ligands brought to the LAT complex, one cannot rule out the potential of redundancy (30, 44). However, this does not appear to be the case, since GRB2 deficiency disrupts LAT clusters. In addition, reconstituting GRB2 deficient cells with wild-type shRNA-resistant GRB2 rescues LAT clustering to a higher degree when compared to LUC control cells. Moreover, a GRB2 with a mutation to the N terminal SH3 domain that eliminates the interaction of GRB2 with SOS1 was incapable of rescuing LAT clusters in GRB2 deficient cells (Figures 6A,B). Overall, our data conclusively show that GRB2 is the key player in the formation of large TCR-induced LAT signaling clusters.

A major unanswered question is what is the clustering of LAT doing to drive TCR-mediated signaling. Interestingly, GRB2 deficient cells had reduced cytoplasmic calcium entry, PLC-γ1 activation, and stable binding of PLC-γ1 to phosphorylated LAT. However, although GRB2 deficient cells had normal levels of LAT Y226 phosphorylation, LAT Y132 had reduced levels of phosphorylation indicating differential regulation of LAT tyrosines by GRB2. The reduction of phosphorylated LAT Y132 in the absence of GRB2 could be a result of the instability of PLC-γ1 at the LAT complex which could be important in shielding phosphorylated Y132 from phosphatases compared to other tyrosines which are bound by GADS or GRAP. Moreover, the disparate results between normal total phosphorylated LAT and phosphorylated Y132 in the absence of GRB2 are likely due to the stoichiometry of phosphorylation between tyrosine residues on LAT. Specifically, LAT Y132 could account for a low percentage of total LAT phosphorylation relative to other sites, and therefore a reduction of 55% of the phosphorylation of Y132 will not be evident in a total phospho-tyrosine blot. Overall, these data and our results strongly suggest the importance of GRB2-induced clustering in stabilizing PLC-γ1 at the LAT-cell membrane junction. The levels of LAT Y132 phosphorylation matched closely with the reduction of PLC-γ1 phosphorylation on Y783 in the absence of GRB2 (Figures 5B,C). These data indicate that Y132 on LAT is required for optimal PLC-γ1 activation. However, the modest reduction of the levels of pY132 on LAT and pY783 on PLC-γ1 in the absence of GRB2 do not explain the undetectable binding between PLC-γ1 and LAT or the substantial impairment of calcium mobilization. Instead, we propose that the binding of GRB2 to Y171, Y191, and Y226 leads to LAT oligomerization that is critical for the stability of PLC-γ1 at the LAT complex. This is supported by previous studies demonstrating that, in addition to PLC-γ1 binding phosphorylated Y132 on LAT, mutation of GRB2-binding tyrosines Y171, Y191, and Y226 inhibited PLC-γ1 recruitment (15, 45).

Next, our results are in contrast to GRB2 haploid insufficiency or complete KO thymocytes where calcium influx was similar or slightly reduced, respectively (17, 18). However, recent GRB2 studies in murine platelets demonstrate that, after GPVI-induced stimulation, GRB2 KO platelets had reduced LAT, SLP-76, and PLC-γ2 phosphorylation and subsequent calcium influx (46). On the other hand, GRB2 can function as a negative regulator of calcium entry in B cells, as GRB2 KO B cells exhibit enhanced proximal signaling and calcium influx (40, 41). Thus, the role of GRB2 in mature human T cells is different relative to thymocytes, B cells, or other hematopoietic cellular signaling pathways. However, in mature human CD4+ T cells, the ability of GRB2 to facilitate LAT clustering is critical for the activation and function of PLC-γ1.

Silencing of GRB2 substantially reduced both protein and transcript levels of IL-2 and IFN-γ in HuT78 T cells. Deficiency of cytokine release was bypassed via PMA-IO stimulation, which suggested a defect at the TCR/LAT complex. However, IL-2 was only partially rescued when GRB2 deficient cells were stimulated through TCR + PMA or IO alone (data not shown). This suggests that the activation of MAP kinases, activated via PMA (47, 48), and calcium influx are needed to fully rescue T cell function in the absence of GRB2. Primary CD4+ T cells from normal human donors also exhibited a significant reduction of TCR-induced IL-2 release. However, we found that TCR-induced IFN-γ was only slightly affected by the knockdown of GRB2 in pre-activated primary CD4+ T cells.

Our results suggest that GRB2 controls TCR-induced IL-2 and IFN-γ through different mechanisms. This idea is supported by our observation that IL-2 was fully rescued while IFN-γ was only partially rescued through PMA-IO stimulation in HuT78 T cells. In addition, basal levels of IFN-γ mRNA in GRB2 deficient cells were not detectable in un-stimulated cells compared to control cells, and only increased slightly after TCR stimulation. The absence of basal IFN-γ mRNA in GRB2 deficient HuT78 T cells indicates regulation of the IFN-γ promoter in T cells via tonic signaling. Finally, GRB2 may differentially regulate cytokine production in T cell lines derived from lymphomas, such as HuT78 T cells, compared to primary T cells. On the basis of these observations, we conclude that GRB2 is required for IL-2 production in T cells, but is only required for TCR-induced IFN-γ secretion in certain T cell populations. Future studies will elucidate if GRB2 is important for differential TCR signaling triggered in naïve versus memory cells, which have distinct capacities to produce IL-2 and IFN-γ (49–51).

In conclusion, our results show that GRB2 is critical for TCR-induced LAT microclusters, calcium influx, and downstream signaling events independent of its ability to negatively regulate signaling at the TCR complex (Figure 8). Importantly, our data suggests that GRB2 KO thymocytes fail to undergo both positive and negative selection as a result of defective LAT-induced signaling observed in GRB2 deficient mature human T cells (17, 18, 52). Results from these studies may also provide further insight into the function of GRB2 downstream of growth factor, cytokine, and insulin receptors (3, 8). These proteins are critical for the initiation and progression of human malignancies and other human diseases (3, 8). These receptors transduce signals in a manner similar to the TCR, although T cells induce signaling through separate kinase and adaptor protein components (5, 10). The paradigm of the field is that GRB2 solely facilitates the recruitment of downstream effector signaling proteins. However, similar to TCR-induced LAT oligomerization, EGF receptors cluster and form supramolecular signaling structures that are critical for signal transduction (53). Collectively, our studies identify a new paradigm of how GRB2 regulates the biology of T cells, but also highlight that GRB2 functions both as an adaptor protein, where it localizes signaling proteins to sites of active signaling, and a structural protein, where it facilitates the clustering of receptors and adaptor proteins.

## Author Contributions

MB conceived and performed the experiments, interpreted data, and wrote the manuscript. JH conceived the experiments, interpreted data, and wrote the manuscript.

## Conflict of Interest Statement

The authors declare that the research was conducted in the absence of any commercial or financial relationships that could be construed as a potential conflict of interest.

## Supplementary Material

The Supplementary Material for this article can be found online at http://journal.frontiersin.org/article/10.3389/fimmu.2015.00141
