# Supplementary figures and images for: GRB2 Nucleates T Cell Receptor-Mediated LAT Clusters That Control PLC-γ1 Activation and Cytokine Production
Source: Front Immunol. 2015 Mar 30;6:141. doi: 10.3389/fimmu.2015.00141 (PMC4378308; doi:10.3389/fimmu.2015.00141)

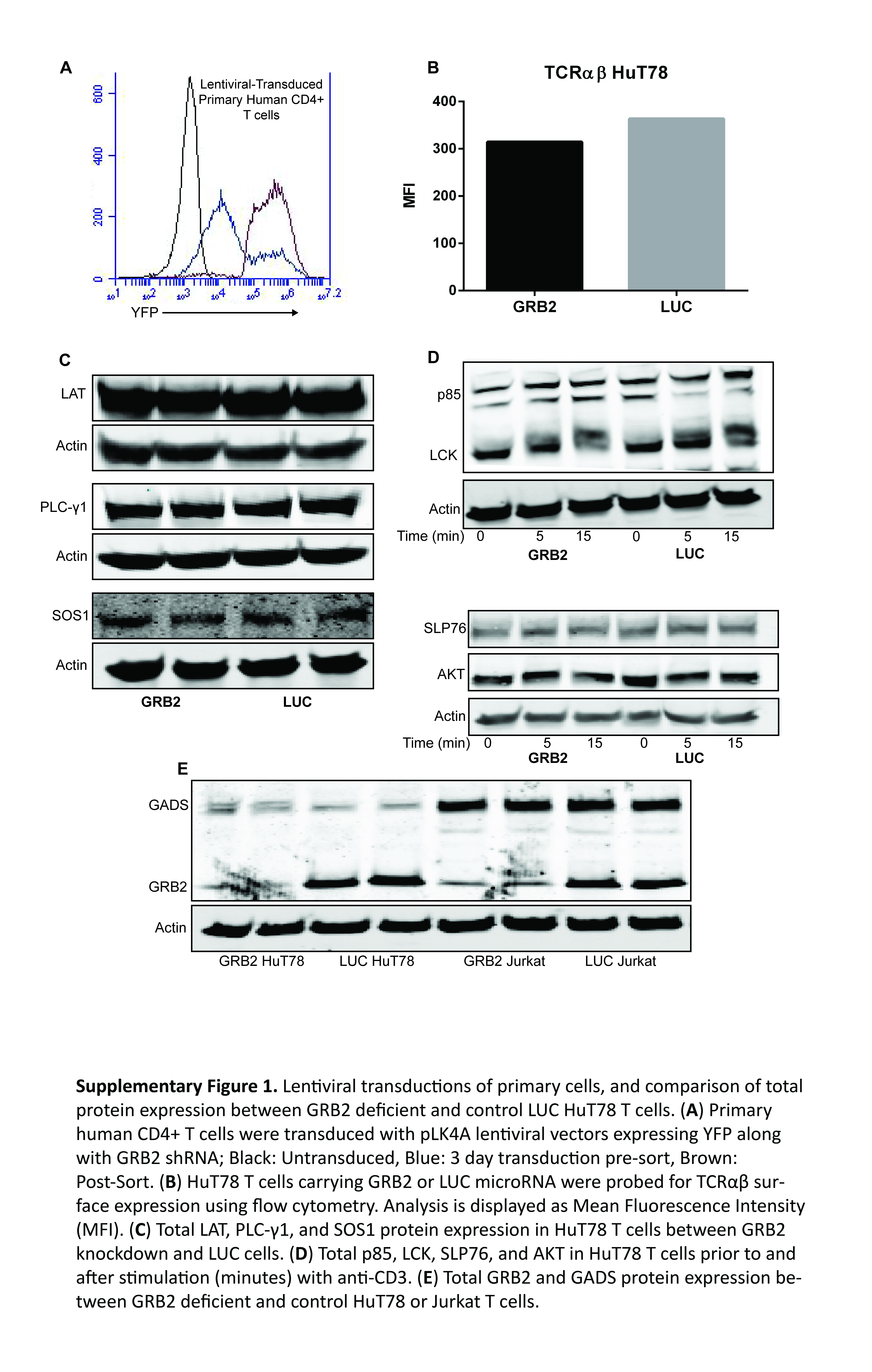

Supplement: Supplementary file 1 [file Image_1.TIF]

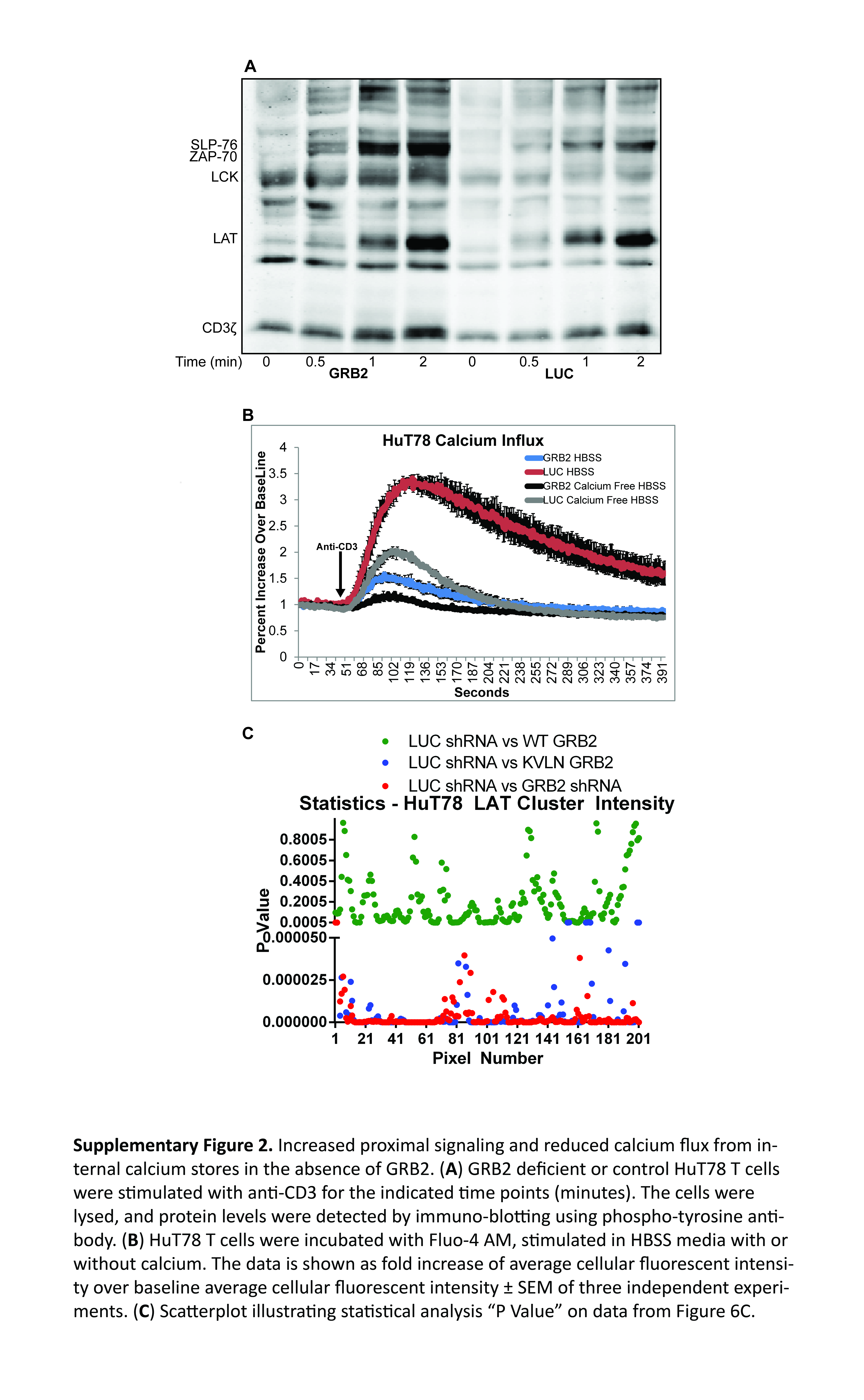

Supplement: Supplementary file 2 [file Image_2.TIF]
